# Influence of Reflective Coating on Temperature Field and Temperature Effect of CRTS III Slab Ballastless Tracks on Bridges

**DOI:** 10.3390/ma16175967

**Published:** 2023-08-31

**Authors:** Li Song, Lei Wu, Chenxing Cui, Zhiwu Yu

**Affiliations:** 1 School of Civil Engineering, Central South University, Changsha 410075, China; songli@csu.edu.cn (L.S.); wulei_199807@163.com (L.W.); zhwyu@csu.edu.cn (Z.Y.); 2 National Engineering Research Center of High-Speed Railway Construction Technology, Changsha 410075, China

**Keywords:** CRTS III slab ballastless track, reflective coating, FEM, temperature field, deformation characteristics, temperature stress

## Abstract

To minimize the adverse effects of high temperatures on the service performance of track structures, research on the application of reflective coatings on track structures is urgently needed. Based on meteorological data and the characteristics of the multi-layer structure of the ballastless track, refined finite element models (FEMs) for the temperature field and temperature effect analysis of the CRTS III slab ballastless track structure on bridges were established. The temperature deformation characteristics and temperature stress distribution of the CRTS III slab ballastless track under natural environmental conditions were investigated. Similarly, the influence of a reflective coating on the structural temperature field and temperature effect was studied. The results showed that the temperature and vertical temperature gradient of the track slab were significantly reduced after the application of the reflective coating. Meanwhile, the thermal deformation and thermal stresses of the track slab and the self-compacting concrete (SCC) layer were minimized. Under high-temperature conditions in summer, the maximum temperature of the track slab decreased from 47.0 °C to 39.6 °C after the application of the reflective coating, and the maximum vertical temperature gradient of the track slab decreased from 61.5 °C/m to 39.1 °C/m after the application of the reflective coating. Under the maximum positive temperature gradient, the peak displacement of the upper arch in the middle of the slab and the peak displacement of the sinking in the slab corner decreased from 0.814 mm and 1.240 mm to 0.441 mm and 0.511 mm, respectively, and the maximum transverse tensile stresses of the track slab reduced from 2.7 MPa to 1.5 MPa as well. In addition, the reflective coating could also inhibit the failure of the interlayer interface effectively. The results of this study can provide a theoretical basis and reference for the application of reflective coatings on ballastless tracks on bridges.

## 1. Introduction

High-speed railways are receiving more and more attention worldwide because of their high stability, high smoothness, and lower maintenance requirements. The Chinese railway track system adopts the CRTS III slab ballastless track structure as the main ballastless track type [1]. The CRTS III slab ballastless track structure was developed by China based on the previous CRTS I and CRTS II ballastless track structures, and it has the characteristics of better integrity and better maintenance. However, due to construction operations, construction conditions, concrete material characteristics, and other factors, the key components of CRTS III slab ballastless tracks are inevitably damaged during service. Therefore, the maintenance of the track structure is essential for the long-term operation of the structure. Due to the poor thermal conductivity of concrete, a ballastless track structure exposed to the environment for a long time is subjected to temperature differences caused by environmental changes, and such temperature differences may lead to deformation, cracking, interlayer separation, and other hazards for the track structure [2,3,4]. These affect the service performance of the track structure. Therefore, it is of great theoretical significance and practical engineering value to investigate the temperature field and temperature effect of CRTS III slab ballastless tracks under temperature loading.

Temperature loads are one of the key loads to be considered in the design of ballastless track structures. Therefore, many scholars in China have studied the structural performance of ballastless tracks under temperature loading. The research approaches mainly include methods based on field measurement results, analytical methods based on heat transfer theory, and methods based on finite element numerical simulation.

Zhao et al. [5] investigated the warpage deformation of CRTS I ballastless tracks in bridge sections under a temperature effect and found that the cement asphalt (CA) quality had a great influence on the force performance of the track structure; meanwhile, the finite element method was used to establish a CRTS I ballastless track model, and the warpage law of the track slab was compared and analyzed by changing the slab thickness and temperature difference conditions. Liu et al. [6] proposed a CRTS II track temperature effect analysis method and studied the mechanical properties of the CA layer and track slab under temperature loading. Song et al. [7] established a defined FEM of the CRTS II ballastless track structure based on meteorological data and the characteristics of the multi-layer structure for ballastless track and elaborated on the characteristics of the thermal deformation, interface damage, and interface separation of CRTS II under natural environmental conditions. Liu et al. [8] established a CRTS III slab ballastless track temperature effect analysis model to study the spatial geometry of the structure and its interface behavior when different layers were connected under natural environmental conditions.

Reflective thermal insulation coatings, as new composite materials that can reduce the absorption coefficient of solar radiation and change the temperature field of a structure, have gradually started to receive attention [9,10,11]. The advantage of a reflective coating is that it can effectively reduce the temperature stress of a track slab and reduce the risk of upper arch deformation [9]; however, its disadvantage is that its use will lead to a sudden drop in the local temperature of the track structure [12], which may have a detrimental effect on the structure. Li et al. [9] experimentally verified the feasibility of reflective coating modeling. In recent years, scholars have studied the type [13] and thickness [14] of the reflective coating, and the results show that a thin layer of a reflective coating on the surface of a structure can effectively reduce the temperature of the structure. Japan and other countries have gradually applied reflective coatings in the military, aviation, and civil construction [15,16,17,18], but their large-scale application on ballastless track structures is still relatively scarce. 

In China, Quan et al. [19] established an FEM for the temperature analysis of CRTS II ballastless tracks on bridges with coatings and verified its validity. Moreover, Quan explored the effect of coatings and coating position on the transverse and longitudinal temperature as well as the temperature gradient of track slabs. Li et al. [9] conducted an experiment on CRTS I double-block ballastless tracks and analyzed the effect of a reflective coating on the temperature field and temperature stress of the track structure under different wind speeds, which provided a reference for the application of reflective coatings in different areas. Liu et al. [12] conducted a full-scale experiment on the CRTS II ballastless track structure on bridges with a reflective coating and investigated the longitudinal stress characteristics of the track slab and base plate before and after the application of a reflective coating; the economy of the reflective coating application position was discussed as well. 

The above studies investigated the effect of solar reflective coatings on the temperature changes of track slabs. However, their research objects were mainly CRTS II slab ballastless tracks, and there has been less research on the effect of reflective coatings on CRTS III slab ballastless tracks; moreover, an analysis of reflective coatings’ impact on the temperature effect in track structures is still lacking. Therefore, the impact of reflective coatings on the temperature effect in the CRTS III slab ballastless track structure was studied in this paper. Based on meteorological data and the characteristics of the track multi-layer structure, refined finite element models of the CRTS III slab ballastless track structure temperature field and temperature effect were established. The effect of reflective coatings on the structural temperature field distribution and temperature effect were investigated; furthermore, the temperature field, temperature deformation characteristics, and temperature stress distribution of CRTS III slab ballastless tracks under natural environmental conditions were revealed. The results of this study can provide a basis for the application of coatings on high-speed railways.

## 2. Method of Temperature Field Analysis

According to the law for the conservation of energy and transformation, without considering the heat generation from the concrete’s internal heat source, the differential equation of thermal conductivity is [20]

(1) $$\frac{\partial T_{i}}{\partial t}=\frac{\lambda_{i}}{\rho_{i}c_{i}}\left(\frac{\partial^{2}T}{\partial x^{2}}+\frac{\partial^{2}T}{\partial y^{2}}+\frac{\partial^{2}T}{\partial z^{2}}\right)\;\;\;\;\;\;\;i=1,\;2,\;3$$

where *T_i_* is the *i*^th^ layer of the track structure, *λ_i_* is the thermal conductivity of the *i*^th^ layer in J/(m∙s∙K), *ρ_i_* is the density of the *i*^th^ layer in kg/m^3^, and *c_i_* is the thermal capacity of the *i*^th^ layer in J/(kg∙K).

### 2.1. Heat Conduction Equation and Boundary Conditions

**Figure 1:**
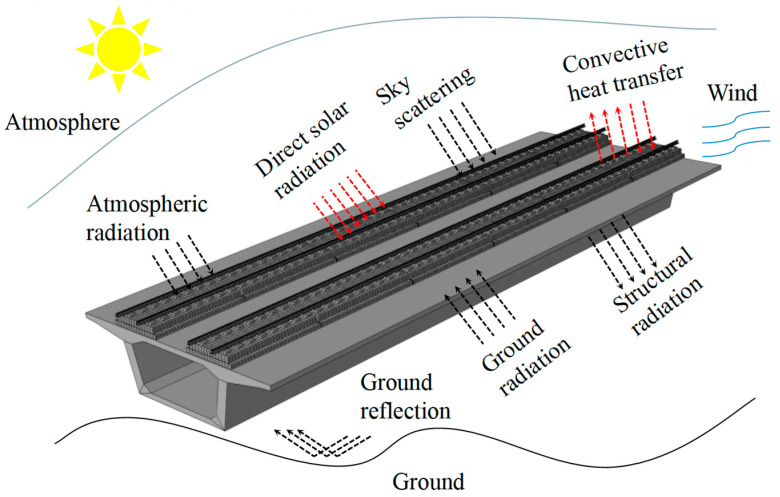
The heat exchange boundary of the CRTS III slab ballastless track structure on the bridge.

The heat exchange boundary of the CRTS III slab ballastless track structure on a bridge is shown in Figure 1. There are three types of heat exchange: solar radiation (short-wave radiation), convective heat transfer, and radiative heat transfer (long-wave radiation), all of which can be converted into the boundary conditions of heat flux, and the total heat flux obtained by the track structure is [20,21]

(2) $$q=q_{s}+q_{c}+q_{r}$$

where *q*_s_ is the heat flux generated by solar radiation on the structure surface (J/(m^2^∙s)); *q*_c_ is the heat flux of convective heat transfer on the structure surface (J/(m^2^∙s)); and *q*_r_ is the heat flux of radiative heat transfer on the structure surface (J/(m^2^∙s)).

#### 2.1.1. Solar Radiation

The solar radiation on the structure’s surface can be divided into three parts: direct radiation, sky scattering, and ground reflection. Because the amount of solar radiation irradiated to the surface of the object surface cannot be completely absorbed, there exists a coefficient *A*_s_ to characterize the ability of the concrete structure to absorb the intensity of solar radiation, and the total heat flux generated by solar radiation on the structure surface can be expressed as [22,23]

(3) $$q_{s}=\left\{\begin{array}{cc} A_{s}\left(I_{d\theta }+I_{d\beta }+I_{dr}\right) & t_{s}\leq t\leq t_{r}\\ 0 & \mathrm{otherwise} \end{array}\right.$$

where *A*_s_ is the absorption rate of concrete, which can be taken as 0.55∼0.70; *A*_s_ is taken according to Ref. [21]; *I_dθ_*, *I_dβ_*, and *I_dr_* are the direct solar radiation intensity, sky scattering intensity, and ground reflection intensity (W/m^2^), respectively; and *t*_s_ and *t*_r_ are the sunrise and sunset times, respectively (h). The above variables are set according to Ref. [7].

#### 2.1.2. Convective Heat Transfer

The heat flux *q*_c_ for convective heat transfer is calculated with the ambient temperature *θ*_a_ and the structure boundary temperature *θ*_γ_ by the following equation, and the heat transfer to the interior of the structure is specified to be positive [24]:

(4) $$q_{c}=h_{c}(\theta_{a}-\theta_{\gamma })$$

where *h*_c_ is the convection coefficient W/(m^2^∙°C), which is related to the shape of the structure boundary, air velocity, ambient temperature, etc. When the wind speed on the concrete surface is *v* ≤ 5.0 m/s, the convection coefficient $h_{c}=2.6\left(\sqrt[{4}]{\theta_{a}-\theta_{\gamma }}+1.54v\right)$. *h*_c_ is set according to Ref. [21].

The ambient temperature *θ*_a_ is calculated by the following equation [25]:

(5) $$\theta_{a}=\left\{\begin{array}{cc} \theta_{\mathrm{ave}}+\theta_{\mathrm{amp}}\cos\left(\frac{\pi }{T}t-\frac{T+T_{\min}}{T}\pi \right) & \left(T_{\min}\leq t\leq T_{\min}+T\right)\\ \theta_{\mathrm{ave}}+\theta_{\mathrm{amp}}\cos\left(\frac{\pi }{24-T}t-\frac{T+T_{\min}}{24-T}\pi \right) & \left(T_{\min}+T\leq t\leq T_{\min}+24\right) \end{array}\right.$$

where *θ*_ave_ is the mean value of daily temperature change (°C); *θ*_amp_ is the amplitude of daily temperature change (°C); *T*_min_ is the time corresponding to the daily minimum temperature (h); and *T*_min_ = 12−0.5(*t*_s_ − *t*_r_); and *T* is the time period corresponding to the change from the minimum temperature to the maximum temperature (h); and *T* = 14 − *T*_min_.

#### 2.1.3. Radiative Heat Transfer

The heat flux *q*_r_ of the radiative heat transfer, the thermal radiation *G_αβ_* received by the structure from the atmosphere, the thermal radiation *U_αβ_* received by the structure from the ground surface, and the intensity *E*_1_ of the structure radiating heat externally are calculated by the following equation [24]:

(6) $$q_{r}=A_{1}\left(G_{\alpha \beta }+U_{\alpha \beta }\right)-E_{1}$$

where *A*_1_ is the material heat radiation absorption rate, which for concrete is generally taken as 0.9, and *G_αβ_* (W/m^2^), *U_αβ_* (W/m^2^), and *E*_1_ (W/m^2^) above are taken according to Ref. [7].

### 2.2. Temperature Field Analysis Model

**Figure 2:**
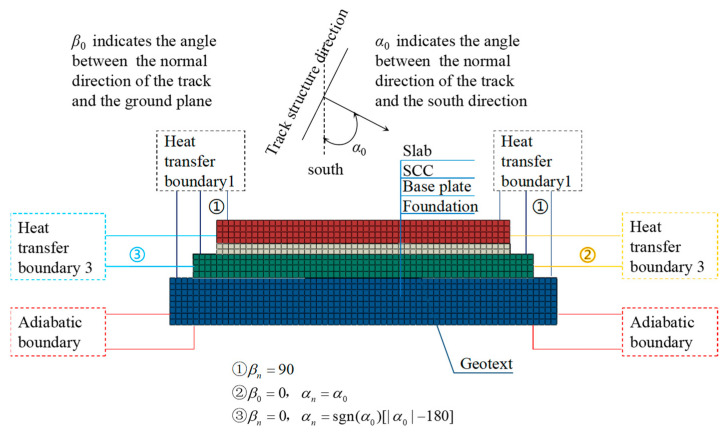
Temperature field analysis model of the CRTS III slab ballastless track structure on bridges.

In this section, a temperature field analysis model of the CRTS III slab ballastless track structure on bridges is established based on the finite element analysis software ABAQUS (Figure 2), with solid element DC3D8 to create the track slab, the SCC, and the base plate. In addition, the thermal resistance effect of the geotextile isolation layer is considered in the model. The model material parameters are shown in Table 1.

**Figure 3:**
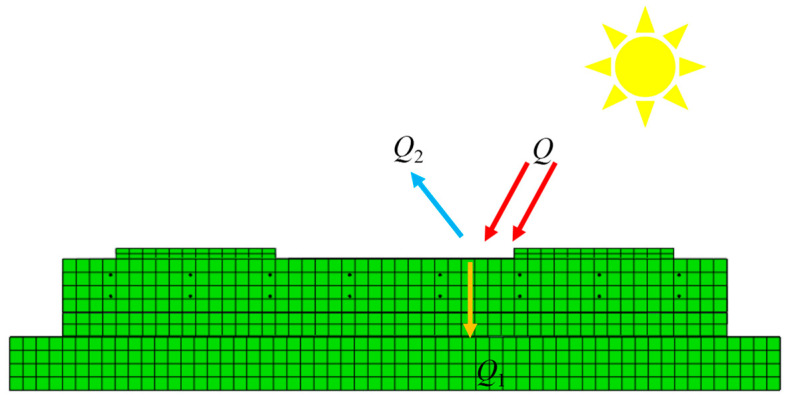
Transmission of solar radiation energy with a reflective coating.

Generally speaking, there are two types of coatings: reflective coatings and barrier coatings. When a structure is exposed to solar radiation, some of the solar radiation is absorbed by the structure and some is reflected [10]. *Q* is the total energy of the structure exposed to solar radiation, *Q*_1_ is the energy absorbed by the structure, and *Q*_2_ is the energy reflected by the structure into the outside world, *Q* = *Q*_1_ + *Q*_2_. Figure 3 presents a schematic diagram for the transmission of solar radiation energy with a reflective coating.

**Figure 4:**
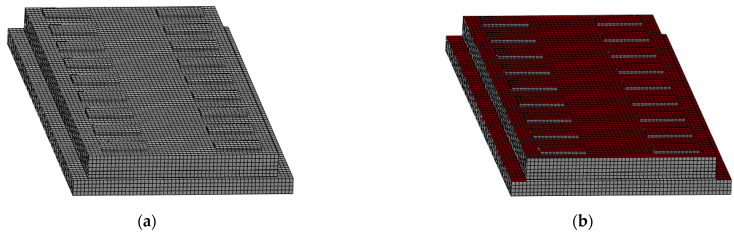
The reflective coating application method: (**a**) before applying the reflective coating; (**b**) after applying the reflective coating.

The coating is mainly composed of a base material and a main material, and the base materials are mostly organic materials such as resin, though sometimes there are also inorganic base materials such as water. The main materials are usually hollow glass beads and vacuum ceramic beads. Reflective coatings are obtained by mixing the base and main material in a mixer in accordance with the appropriate specifications [10]. The thickness of a reflective coating is usually 5–20 mm [19], and the thickness of the coating is small relative to the size of the structure. Therefore, the reflective coating is neglected in the modeling, and only the effect of coating on the solar radiation absorption coefficient of the concrete is considered. In this paper, the parameters of the reflective coating in Ref. [19] are used: the solar radiation absorption coefficient of the coating is 0.37, and the emission coefficient is 0.82. In the calculation, the solar radiation is multiplied by the corresponding absorption coefficient and added to the ballastless track surface as a heat flux load. Figure 4 shows the reflective coating application method. It can be seen from Figure 4 that the temperature field analysis process of the CRTS III slab ballastless track structure with the coating is the same as in Section 2.3, and the corresponding model material parameters are the same as in Section 2.2.

### 2.3. Process of Temperature Field Analysis 

**Figure 5:**
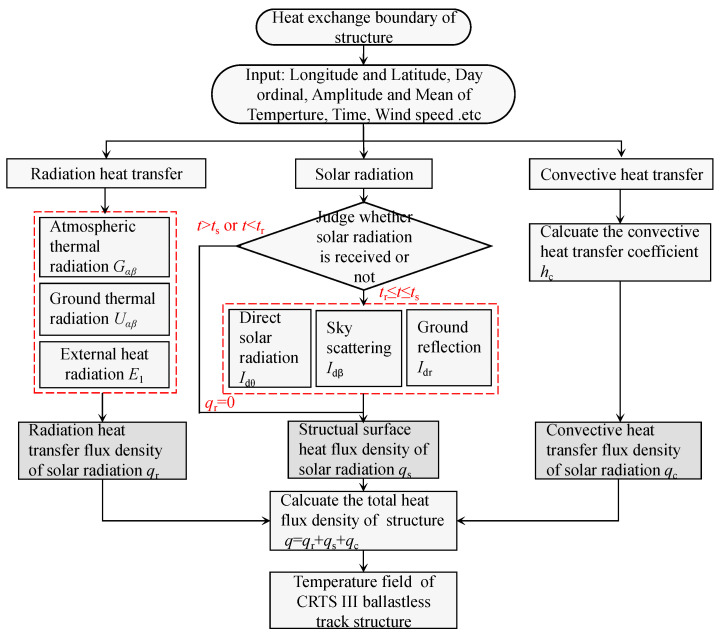
Calculation process of temperature field for the CRTS III slab ballastless track structure on a high-speed railway bridge.

The calculation process of the temperature field for the CRTS III slab ballastless track structure on a high-speed railway bridge is shown in Figure 5.

The calculation process of the temperature field for the CRTS III slab ballastless track structure on a high-speed railway bridge is as follows (Figure 5): Input the latitude and longitude of the region and day ordinal to determine the solar constant, solar declination, solar altitude angle, and other relevant physical parameters according to the formula in Section 2.1. Then, combine the measured temperature data to determine the daily temperature mean value *θ*_ave_ and the daily temperature amplitude *θ*_amp_, and calculate *q*_s_, *q*_r_, and *q*_c_, respectively. Finally, calculate the total heat flux *q* and bring it into the FEM as the boundary condition of the structure. Then, calculate the temperature field of the CRTS III slab ballastless track structure on high-speed railway bridges.

### 2.4. Model Validation 

**Figure 6:**
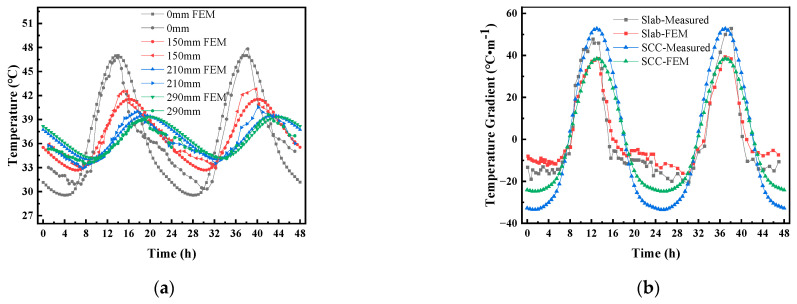
Comparison of the measured and calculated temperature values at different depths in the middle of the track slab: (**a**) temperature variation of track slab; (**b**) temperature gradient variation of track slab.

According to Ref. [8], the corresponding meteorological data of Changsha (28°8′22″ N, 112°59′28″ W) from 25 to 26 July 2019 were selected. The average values of temperature for 25 and 26 July were 32.5 °C and 33.5 °C, respectively, and the temperature amplitude was 3.5 °C for both. We input the data into the temperature analysis model proposed in Section 2.2. Figure 6 shows the comparison between the measured and calculated temperature values at different depths in the middle of the track slab (the value in millimeters indicates the vertical distance of the point from the top of the track slab). It can be seen from Figure 6 that the simulated results and the measured temperature were consistent and showed the same trend, so the temperature field analysis model of the CRTS III slab ballastless track structure based on meteorological data proposed in this paper is accurate and reliable.

## 3. Temperature Field of CRTS III Slab Ballastless Track

### 3.1. Vertical Temperature and Temperature Gradient

Taking the Nanjing area (32°02′38″ N, 118°46′43″ E) as an example, the meteorological parameters of the area on 15 July 2014 were selected. The method of calculating the temperature field proposed in Section 2.3 of this paper was used to analyze the temperature field of CRTS III slab ballastless track under natural environmental conditions, and the ballastless track structure and materials were consistent with those in Section 2.2. 

**Figure 7:**
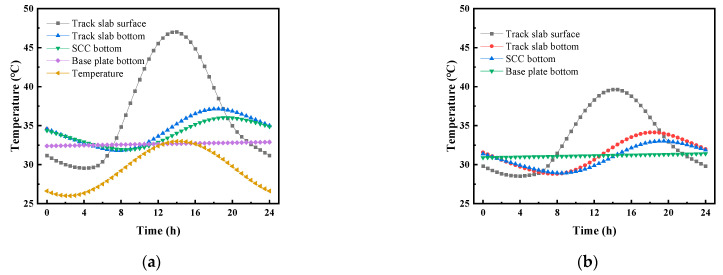
The daily distribution curve for the vertical temperature of the CRTS III slab ballastless track structure on 15 July 2014: (**a**) vertical temperature; (**b**) vertical temperature after coating.

Figure 7 shows the daily distribution curve for the vertical temperature of the CRTS III slab ballastless track structure before and after the application of a reflective coating on 15 July 2014. Figure 7 shows that the average temperature on 15 July 2014 was 29.4 °C, and the corresponding maximum temperature of 32.9 °C and minimum temperature of 25.9 °C occurred at approximately 14:30 and 03:30, respectively. With the daily change in atmospheric temperature, the temperature of the track structure showed different temperature response laws. After applying the reflective coating, the temperature change of the track slab was the most obvious. The highest temperature of the track slab surface appeared at 14:00 and decreased from 47.0 °C to 39.6 °C; the lowest temperature of the track slab surface appeared at 3:30 and decreased from 29.6 °C to 28.5 °C. It can be seen that the reflective coating had a greater effect on the highest temperature of the structure and had little effect on the lowest temperature of the structure. After applying the reflective coating, the maximum temperature of the SCC layer appeared at 14:00 and decreased from 37.1 °C to 34.1 °C; the minimum temperature of the SCC layer appeared at 3:30 and decreased from 31.9 °C to 28.8 °C. The maximum temperature of the base plate appeared at 14:00 and decreased from 36.0 °C to 33.0 °C; the lowest temperature of the base plate appeared at 3:30 and decreased from 31.9 °C to 28.8 °C. 

**Figure 8:**
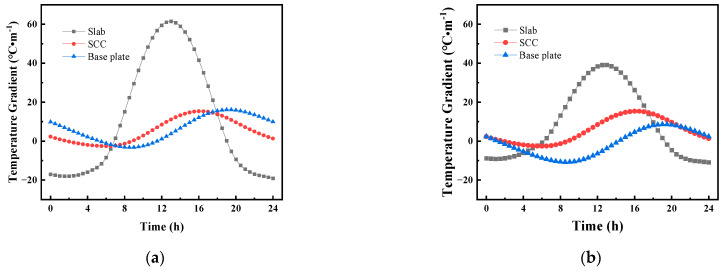
The daily distribution curve for the vertical temperature gradient of the CRTS III slab ballastless track structure on 15 July 2014: (**a**) vertical temperature gradient; (**b**) vertical temperature gradient after coating.

Figure 8 shows the daily distribution curve for the vertical temperature gradient of the CRTS III slab ballastless track structure before and after the application of the reflective coating on 15 July 2014. Figure 8 shows that the vertical temperature gradient of the track structure varied periodically with time, and the temperature gradient within the track slab changed most significantly after the application of the reflective coating. After applying the reflective coating, the highest temperature gradient of the track slab appeared at 14:00 and decreased from 61.5 °C/m to 39.1 °C/m; the lowest temperature gradient of the track slab appeared at 3:30 and decreased from −18.0 °C/m to −9.1 °C/m. After applying the reflective coating, the highest temperature gradient of the SCC appeared at 14:00 and decreased from 15.4 °C/m to 15.3 °C/m; the lowest temperature gradient of the SCC appeared at 3:30 and decreased from −2.5 °C/m to −2.6 °C/m. The highest temperature gradient of the base plate appeared at 20:00 and decreased from 16.1 °C/m to 8.6 °C/m; the lowest temperature gradient of the base plate appeared at 09:30 and decreased from −3.2 °C/m to −10.8 °C/m.

### 3.2. Transverse Temperature and Temperature Gradient

**Figure 9:**
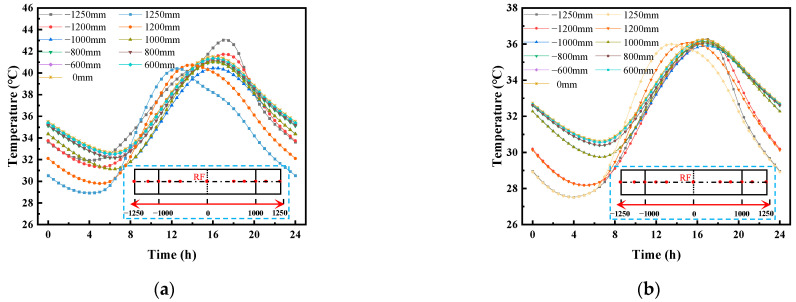
The daily distribution curve for the transverse temperature of the CRTS III slab ballastless track structure on 15 July 2014: (**a**) transverse temperature; (**b**) transverse temperature after coating.

Figure 9 shows the daily distribution curve for the transverse temperature of the CRTS III slab ballastless track structure before and after the application of the reflective coating on 15 July 2014. It is stipulated that the reference plane was along the transverse middle section and that the right side and the left side were positive (sunny side) and negative (negative side), respectively. As can be seen from Figure 9, the temperature variation of the track slab was most obvious within the range of 250 mm on both sides of the track slab, and the temperature changes in other areas of the track slab were relatively moderate. After applying the reflective coating, the highest lateral temperature of the track slab decreased from 43.03 °C to 36.25 °C; the lowest temperature decreased from 28.91 °C to 27.50 °C.

**Figure 10:**
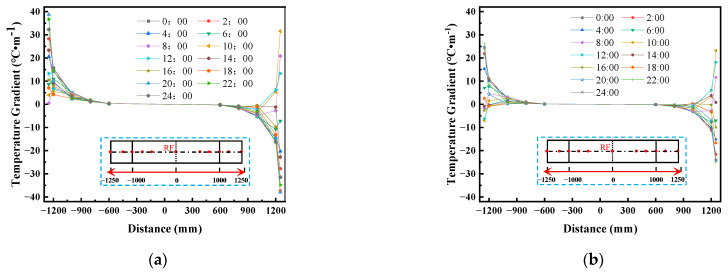
Transverse temperature gradient curve for the track slab at specific moments on 15 July 2014: (**a**) transverse temperature gradient; (**b**) transverse temperature gradient after coating.

Figure 10 shows the transverse temperature gradient curve for the track slab at specific moments before and after the application of the reflective coating on 15 July 2014. As can be seen from Figure 10, the highest transverse temperature gradient of the track slab decreased from 38.7 °C/m to 26.3 °C/m; the lowest transverse temperature gradient decreased from −38.0 °C/m to −24.3 °C/m.

## 4. Analysis Method of Temperature Effect

### 4.1. Finite Element Model of CRTS III Slab Ballastless Track

**Figure 11:**
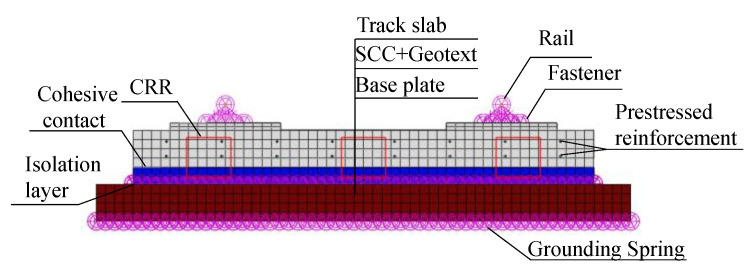
A defined finite element model of the CRTS III slab ballastless track structure.

In this section, a defined finite element model of the CRTS III slab ballastless track structure is established (Figure 11), which includes the rail fastener system, the precast track slab, the cohesive contact between the slab and the SCC, the SCC, and the base plate. In addition, springs are used to simulate the interlayer interaction between the slab and the SCC and the effect of the foundation on the support of the base plate. The structural material parameters of the CRTS III slab ballastless track are shown in Table 2.

#### 4.1.1. Fastener System

**Figure 12:**
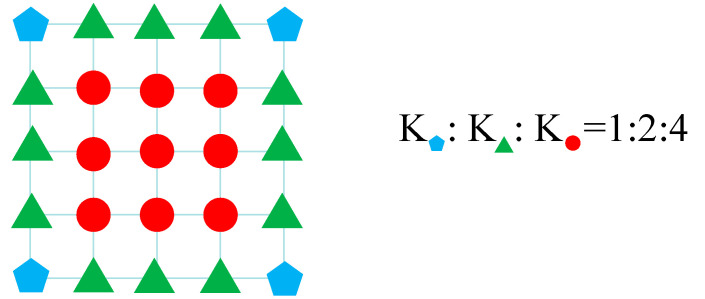
The schematic diagram of the fastener stiffness distribution.

To reduce the stress concentration phenomenon caused by a single spring connection, the fastener system is equated to 25 space spring units, as shown in Figure 12. Based on Winkler’s assumption of the energy equivalence principle, the pentagon is 1/64 of the total fastener stiffness, the triangle is 1/32 of the total fastener stiffness, and the circle is 1/16 of the total fastener stiffness. The vertical stiffness and transverse stiffness are 50 kN/mm and 35 kN/mm, respectively [26], and the longitudinal stiffness is related to the longitudinal displacement, which is calculated according to Table 3.

#### 4.1.2. Precast Track Slab

The track slab of the CRTS III slab ballastless track structure is a two-way prestressed structure, and this paper uses the prestressing field to apply the prestressing. In ABAQUS, the prestressing steel bars are modeled by solid units, and the prestressing steel bars are embedded in the track slab to establish the nodal degrees of freedom coupling between the track slab’s solid units and the prestressing steel bars’ solid units. In this paper, spiral ribbed steel bars with a nominal diameter of 10 mm are used as the prestressing steel bars, and the prestressed analysis model of the CRTS III track slab is established by ABAQUS based on the unit embedding technique. The prestress states are obtained by the stress field method, and the loss of prestressing force is considered according to Ref. [27]. After the loss of the prestressing force, the transverse and longitudinal prestress in the track slab are 836 MPa and 843 MPa, respectively.

#### 4.1.3. Interlayer Contact Model

**Figure 13:**
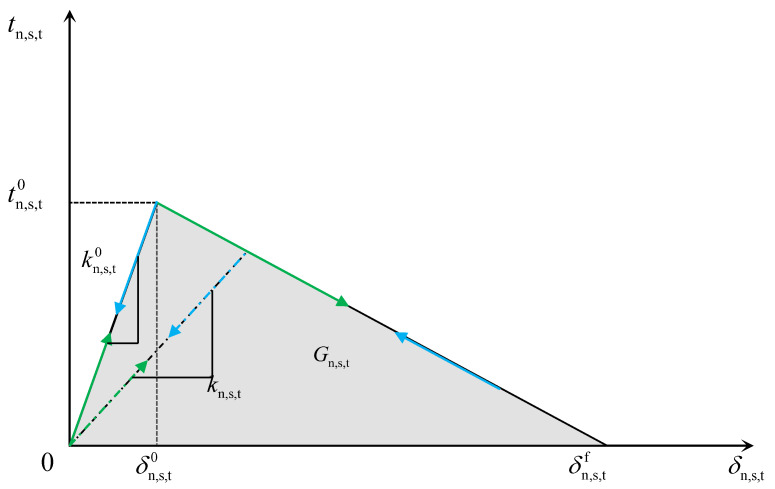
The bi-linear cohesive principal structure model.

The cohesive surface behavior is used to model the bonding interface between the track slab and the SCC, and the bi-linear cohesive principal model is chosen to characterize its behavior, as shown in Figure 13 [28].

The damage starts when the quadratic interaction function of the contact stress ratio reaches 1, and the damage onset can be expressed as [28]

(7) $${\left\{\left.\frac{\left(t_{n}\right)}{t_{n}^{0}}\right\}\right.}^{2}+{\left\{\left.\frac{t_{s}}{t_{s}^{0}}\right\}\right.}^{2}+{\left\{\left.\frac{\left(t_{t}\right)}{t_{t}^{0}}\right\}\right.}^{2}=1$$

where $t_{n}^{0}$, $t_{s}^{0}$, and $t_{t}^{0}$ denote the critical stress in normal and two-shear directions, respectively.

In order to characterize the evolution of damage under the combination of normal and shear separation at the interface, an effective separation *δ*_m_ is introduced [28]:

(8) $$\delta_{m}=\sqrt{{\left(\delta_{n}\right)}^{2}+\delta_{s}^{2}+\delta_{t}^{2}}$$

The damage variable *D* represents the overall damage at the contact point, which initially has a value of 0 and evolves monotonically from 0 to 1 at further loading after the onset of damage. The contact stress components can be expressed in Equation (9) through Equation (12); ABAQUS uses the damage variable *D* to characterize the interlaminar damage, and *D* can be expressed as follows [28]:

(9) $$t_{n}=\left\{\begin{array}{cc} \left(1-D\right){\bar{t}}_{n} & {\bar{t}}_{n}\geq 0\\ {\bar{t}}_{n} & \mathrm{otherwise} \end{array}\right.$$

(10) $$t_{s}=\left(1-D\right){\bar{t}}_{s}$$

(11) $$t_{t}=\left(1-D\right){\bar{t}}_{t}$$

(12) $$D=\frac{\delta_{m}^{f}\left(\delta_{m}^{\max}-\delta_{m}^{0}\right)}{\delta_{m}^{\max}\left(\delta_{m}^{f}-\delta_{m}^{0}\right)}$$

where *t*_n_, *t*_s_, and *t*_t_ denote the contact stress component; $\delta_{m}^{\max}$ is the maximum value of effective separation reached during loading; and $\delta_{m}^{f}$ and $\delta_{m}^{0}$ are the effective displacements at damage initiation and fracture, respectively, in the mixed mode.

The interface failure criterion can be expressed as [28]

(13) $${\left\{\left.\frac{G_{n}}{G_{n}^{c}}\right\}\right.}^{2}+{\left\{\left.\frac{G_{s}}{G_{s}^{c}}\right\}\right.}^{2}+{\left\{\left.\frac{G_{t}}{G_{t}^{c}}\right\}\right.}^{2}=1$$

where *G*_n_, *G*_s_, and *G*_t_ denote the fracture energy under load, and $G_{n}^{c}$, $G_{s}^{c}$, and $G_{t}^{c}$ denote the critical fracture energy.

In addition, the geotextile isolation layer between the SCC and the base plate is simulated by a nonlinear spring unit with a stiffness of 500 MPa/m, and the elastic buffer layer in the groove of the base in the transverse and longitudinal directions is simulated by linear springs with a stiffness of 54 MPa/m and 50 MPa/m, respectively.

### 4.2. Framework of Temperature Effect Analysis

**Figure 14:**
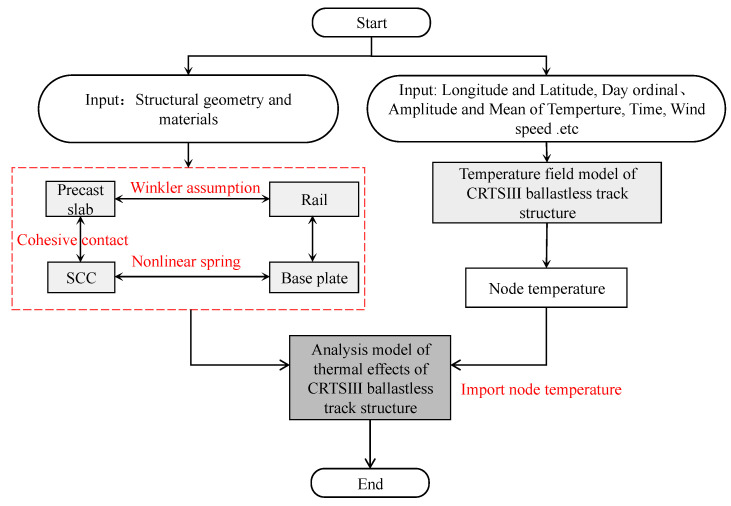
Calculation process of thermal effect analysis for the CRTS III slab ballastless track structure.

Based on the temperature field model and temperature effect analysis model of the CRTS III slab ballastless track structure proposed earlier, a temperature effect analysis method for the CRTS III slab ballastless track structure under natural environmental conditions is proposed. The flow chart is shown in Figure 14.

The calculation process of the thermal effect analysis for the CRTS III slab ballastless track structure is as follows (Figure 14):

1. Model the temperature field of the CRTS III slab ballastless track structure under natural environmental conditions, as in Section 2.2;
2. Establish an analytical model of the thermal effects of the CRTS III slab ballastless track structure considering the rails, track slab, interlayer contact, and other components, as in Section 4.1;
3. Import the temperature field calculated in (1) into the thermal effect analysis model for the CRTS III slab ballastless track structure in (2) for thermal coupling analysis, and finally derive the thermal effect of the CRTS III slab ballastless track structure under natural environmental conditions.

### 4.3. Method Validation

**Figure 15:**
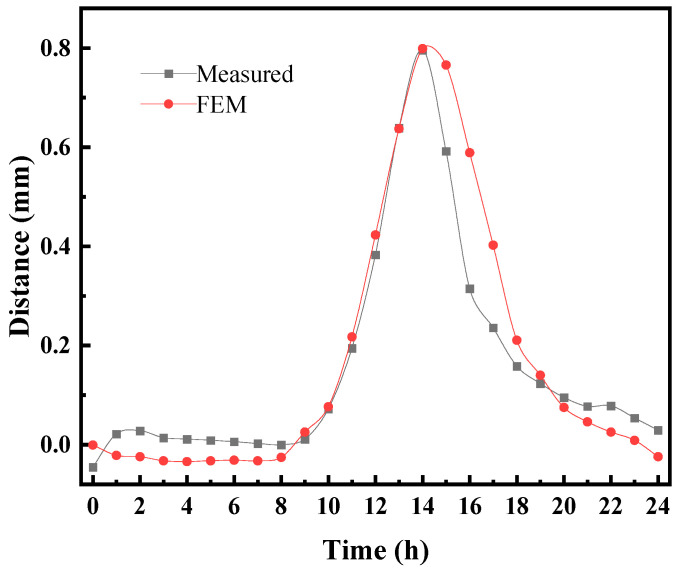
Comparison of measured displacement and simulation.

To verify the reliability of the temperature effect analysis of the CRTS III slab ballastless track under natural environmental conditions, the temperature field in Figure 6 was imported into the temperature effect analysis model, and then the simulated results were compared with the experimental results in Ref. [8]. Figure 15 shows the comparison between the vertical displacement obtained from the experiment and the simulation in the track slab. It can be seen from Figure 15 that the vertical displacement obtained from the experiment at 13:00 and the simulated vertical displacement were 0.798 mm and 0.801 mm, respectively. The error of the simulated results was smaller compared with that of the experimental results, which verified the accuracy of the model. 

## 5. Temperature Effect of CRTS III Slab Ballastless Track

### 5.1. Track Slab

The warpage deformation of a track slab under the temperature gradient affects the normal service of the track structure, so it is necessary to study the effect of applying a reflective coating on the mechanical properties of the structure. Under the positive temperature gradient, the structure showed a convex spatial deformation pattern: sinking in the corner of the slab and arching in the middle of the slab.

**Figure 16:**
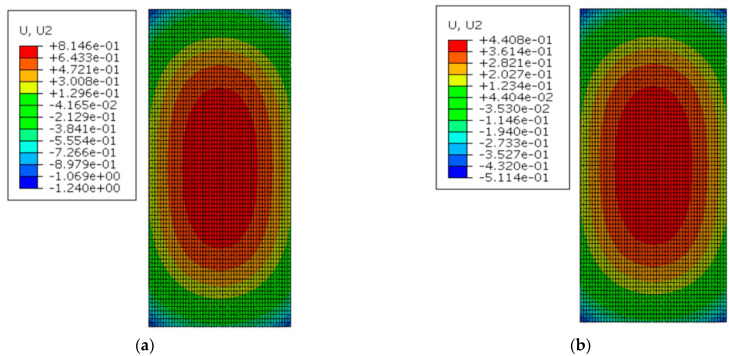
The deformation of the track slab under the maximum positive temperature gradient (mm): (**a**) before coating; (**b**) after coating.

Figure 16 shows the deformation of the track slab under the maximum positive temperature gradient before and after coating. As can be seen from Figure 16, the peak displacement of the upper arch in the middle of the track slab decreased from 0.814 mm to 0.441 mm with the reflective coating; meanwhile, the peak displacement of the sinking in the track slab corner decreased from 1.240 mm to 0.511 mm after applying the reflective coating.

**Figure 17:**
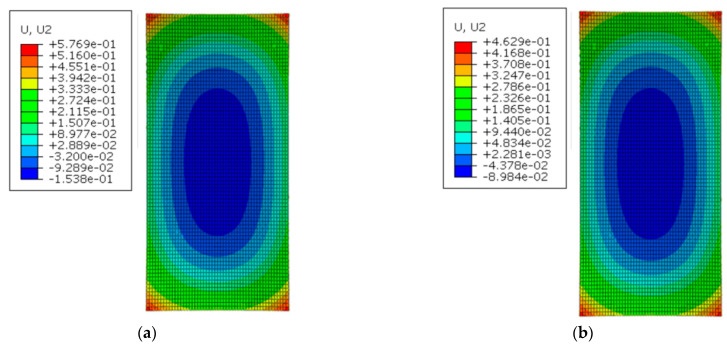
The deformation of the track slab under the maximum negative temperature gradient (mm): (**a**) before coating; (**b**) after coating.

Figure 17 shows the deformation of the track slab under the maximum negative temperature gradient before and after coating. Under the negative temperature gradient, the structure showed concave deformation: warping in the slab corner, and concave deformation in the middle of the slab. As can be seen from Figure 17, the peak displacement of sinking in the middle of the track slab decreased from 0.154 mm to 0.090 mm with the reflective coating; meanwhile, the peak displacement of the upward arch in the track slab corner decreased from 0.577 mm to 0.463 mm with the reflective coating.

Warp tensile stress is generated inside a track slab under the temperature gradient. However, the concrete’s tensile strength is low, so the warp tensile stress of the track slab is a key concern in the design of the track structure.

**Figure 18:**
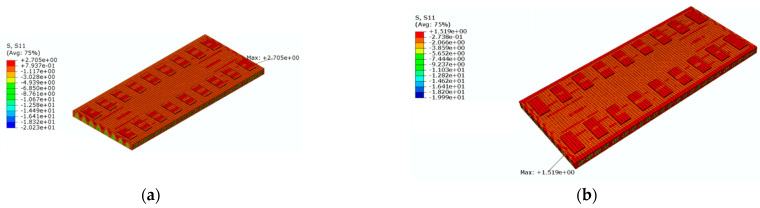
The cloud diagram for the maximum transverse tensile stress s11 of the track slab under the maximum positive temperature gradient (MPa): (**a**) before coating; (**b**) after coating.

Figure 18 shows a cloud diagram for the maximum transverse tensile stress s11 of the track slab under the maximum positive temperature gradient before and after coating. As can be seen from Figure 18, the maximum tensile stress in the transverse direction of the track slab appeared on the outer surface of the track slab in the transverse direction. Under the maximum positive temperature gradient, the maximum tensile stress s11 in the transverse direction of the track slab decreased from 2.7 MPa to 1.5 Mpa after the reflective coating was applied. 

**Figure 19:**
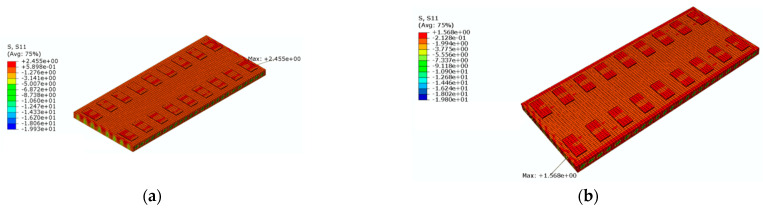
The cloud diagram for the maximum transverse tensile stress s11 of the track slab under the maximum negative temperature gradient (MPa): (**a**) before coating; (**b**) after coating.

Figure 19 shows a cloud diagram for the maximum transverse tensile stress s11 of the track slab under the maximum negative temperature gradient before and after coating. As can be seen from Figure 19, the maximum tensile stress in the transverse direction of the track slab appeared on the outer surface of the track slab in the transverse direction. Under the maximum negative temperature gradient, the maximum tensile stress s11 in the transverse direction of the track slab decreased from 2.5 MPa to 1.6 MPa after the reflective coating was applied. It can be seen that the reflective coating could significantly reduce the maximum transverse stress in the track slab under the maximum positive and negative temperature gradients.

**Figure 20:**
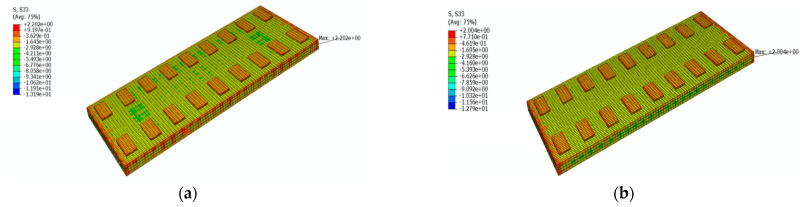
The cloud diagram for the maximum longitudinal tensile stress s33 of the track slab under the maximum positive temperature gradient (MPa): (**a**) before coating; (**b**) after coating.

Figure 20 shows a cloud diagram for the maximum longitudinal tensile stress s33 of the track slab under the maximum positive temperature gradient before and after coating. As can be seen from Figure 20, the maximum tensile stress in the longitudinal direction of the track slab appeared on the outer surface of the track slab in the longitudinal direction. Under the maximum positive temperature gradient, the maximum tensile stress s33 in the longitudinal direction of the track slab decreased from 2.2 Mpa to 2.0 Mpa after the reflective coating was applied.

**Figure 21:**
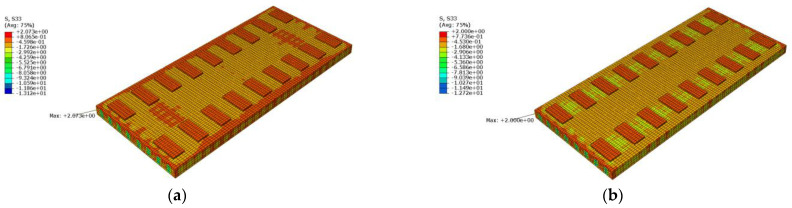
The cloud diagram for the maximum longitudinal tensile stress s33 of the track slab under the maximum negative temperature gradient (MPa): (**a**) before coating; (**b**) after coating.

Figure 21 shows a cloud diagram for the maximum longitudinal tensile stress s33 of the track slab under the maximum negative temperature gradient before and after coating. As can be seen from Figure 21, the maximum tensile stress in the longitudinal direction of the track slab appeared on the outer surface of the track slab in the longitudinal direction. Under the maximum negative temperature gradient, the maximum tensile stress s33 in the longitudinal direction of the track slab decreased from 2.1 Mpa to 2.0 Mpa after the reflective coating was applied. The reflective coating was not effective in reducing the maximum longitudinal tensile stress in the track slab under the maximum positive and negative temperature gradients, which was caused by the high number of longitudinal prestressing steel bars in the track slab.

### 5.2. SCC

The SCC layer is an important part of the CRTS III slab ballastless track structure, and it is also the weakest part of the track structure. Thus, the analysis of vertical displacement and maximum tensile stress in the longitudinal and transverse directions of the SCC is necessary.

**Figure 22:**
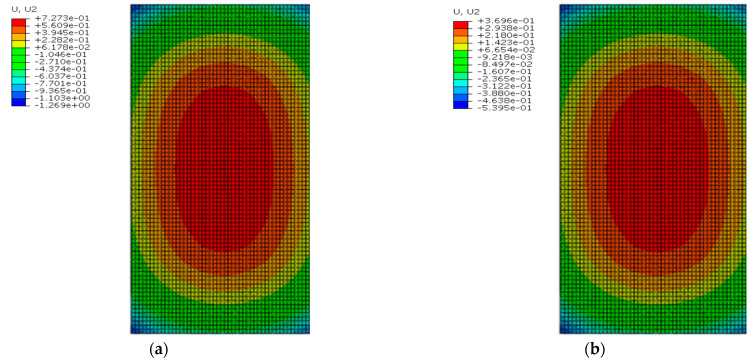
The deformation of the SCC under the maximum positive temperature gradient (mm): (**a**) before coating; (**b**) after coating.

Figure 22 shows the deformation of the SCC under the maximum positive temperature gradient before and after coating. As can be seen from Figure 22, the SCC also showed a convex deformation like the track slab under the positive temperature gradient. The peak displacement of the upper arch in the middle of the SCC decreased from 0.727 mm to 0.370 mm with the reflective coating; meanwhile, the peak displacement of the sinking in the SCC corner decreased from 1.269 mm to 0.540 mm with the reflective coating.

**Figure 23:**
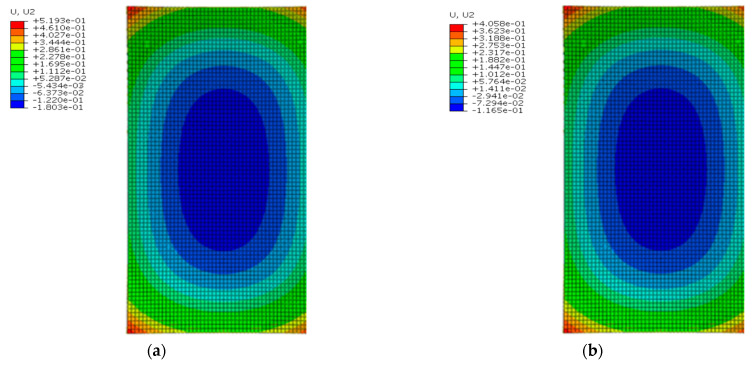
The deformation of the SCC under the maximum negative temperature gradient (mm): (**a**) before coating; (**b**) after coating.

Figure 23 shows the deformation of the SCC under the maximum negative temperature gradient before and after coating. As can be seen from Figure 23, the SCC also showed a concave deformation like the track slab under the negative temperature gradient. The peak displacement of sinking in the middle of SCC decreased from 0.180 mm to 0.117 mm with the reflective coating; meanwhile, the peak displacement of the upward arch of the SCC corner decreased from 0.519 mm to 0.406 mm with the reflective coating.

**Figure 24:**
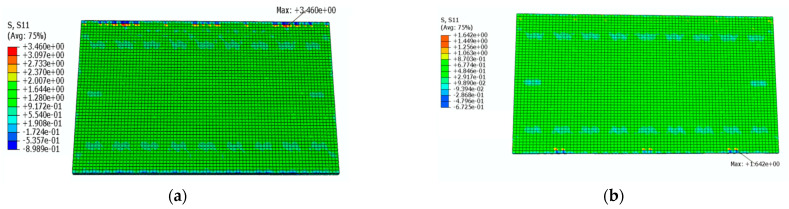
The cloud diagram for the maximum transverse tensile stress s11 of the SCC under the maximum positive temperature gradient (MPa): (**a**) before coating; (**b**) after coating.

Figure 24 shows a cloud diagram for the maximum transverse tensile stress s11 of the SCC under the maximum positive temperature gradient before and after coating. As can be seen from Figure 24, the maximum transverse tensile stress of the SCC occurred at the upper surface of the SCC. Under the maximum positive temperature gradient, the maximum tensile stress s11 in the transverse direction of the SCC decreased from 3.5 MPa to 1.6 Mpa after the reflective coating was applied.

**Figure 25:**
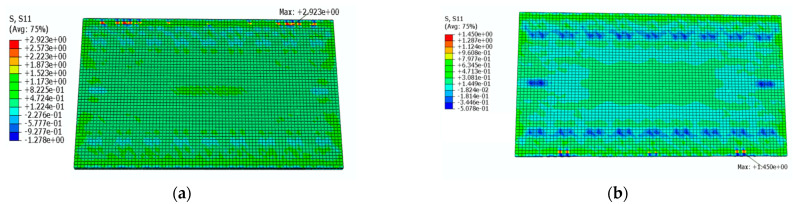
The cloud diagram for the maximum transverse tensile stress s11 of the SCC under the maximum negative temperature gradient (MPa): (**a**) before coating; (**b**) after coating.

Figure 25 shows a cloud diagram for the maximum transverse tensile stress s11 of the SCC under the maximum negative temperature gradient before and after coating. As can be seen from Figure 25, the maximum transverse tensile stress of the SCC occurred at the upper surface of the SCC. Under the maximum negative temperature gradient, the maximum tensile stress s11 in the transverse direction of the SCC decreased from 2.9 Mpa to 1.5 Mpa after the reflective coating was applied. It can be seen that the reflective coating could significantly reduce the maximum transverse tensile stress in the SCC layer under the maximum positive and negative temperature gradients.

**Figure 26:**
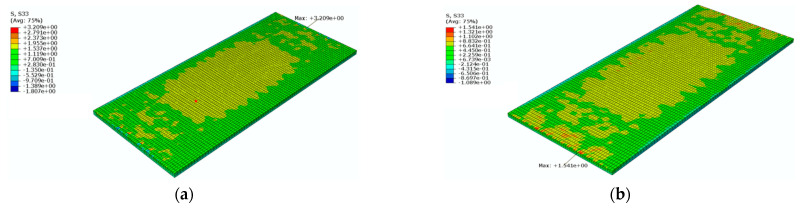
The cloud diagram for the maximum longitudinal tensile stress s33 of the SCC under the maximum positive temperature gradient (MPa): (**a**) before coating; (**b**) after coating.

Figure 26 shows a cloud diagram for the maximum longitudinal tensile stress s33 of the SCC under the maximum positive temperature gradient before and after coating. As can be seen from Figure 26, the maximum longitudinal tensile stress of the SCC occurred at the upper surface of the SCC. Under the maximum positive temperature gradient, the maximum tensile stress s33 in the longitudinal direction of the SCC decreased from 3.2 Mpa to 1.5 Mpa after the reflective coating was applied.

**Figure 27:**
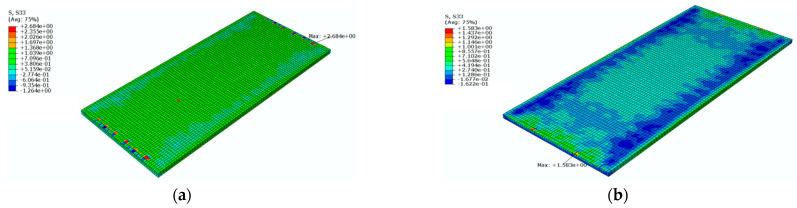
The cloud diagram for the maximum longitudinal tensile stress s33 of the SCC under the maximum negative temperature gradient (MPa): (**a**) before coating; (**b**) after coating.

Figure 27 shows a cloud diagram for the maximum longitudinal tensile stress s33 of the SCC under the maximum negative temperature gradient before and after coating. As can be seen from Figure 27, the maximum longitudinal tensile stress of the SCC occurred at the upper surface of the SCC. Under the maximum negative temperature gradient, the maximum tensile stress s33 in the longitudinal direction of the SCC decreased from 2.7 Mpa to 1.6 Mpa after the reflective coating was applied. It can be seen that the reflective coating could significantly reduce the maximum longitudinal tensile stress in the SCC layer under the maximum positive and negative temperature gradients.

The reflective coatings work by lowering the temperature of the structure, which in turn affects the forces on the track structure and ultimately reduces the thermal stresses and thermal deformation of the track structure.

### 5.3. Interface Damage

The SCC layer is an important part of the CRTS III slab ballastless track structure, and it is the weakest part of the track structure. Under a higher temperature gradient, the interlayer interface of the track structure is prone to damage, and this will affect the service performance of the structure, so the mechanical performance of the interlayer interface is the key concern in the service process of the track slab. In ABAQUS, CSQUADCRT is the cohesive contact initiation damage parameter, and the interlayer interface only starts to be damaged when CSQUADCRT is equal to 1. CSDMG is the cohesive contact damage parameter, meaning that the interlayer interface starts to be damaged when CSDMG is greater than 0; the interlayer interface can be considered to be completely failed when CSDMG reaches 1.

**Figure 28:**
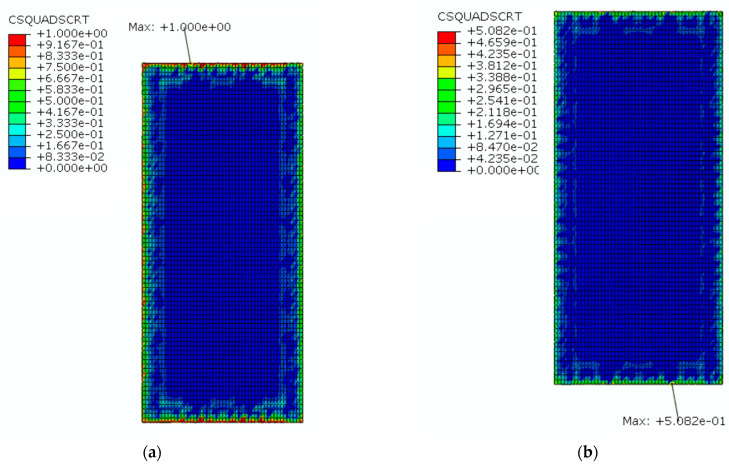
The cloud diagram for the interlaminar interface initiation damage parameters: (**a**) before coating; (**b**) after coating.

Figure 28 shows a cloud diagram for the interlaminar interface initiation damage parameters before and after coating. From Figure 28, it can be seen that the starting crack of the interlaminar interface was initiated from the edge of the interlaminar interface. Before the reflective coating was applied, the maximum value of the structure’s CSQUADCRT under natural environmental conditions for one day reached 1, and the interlaminar interface started to be damaged. After the reflective coating was applied, the maximum value of the structure’s CSQUADCRT under natural environmental conditions for one day was less than 1 and had not yet reached the starting damage value of the interlaminar interface, so the interlaminar interface had not begun to be damaged at this time.

**Figure 29:**
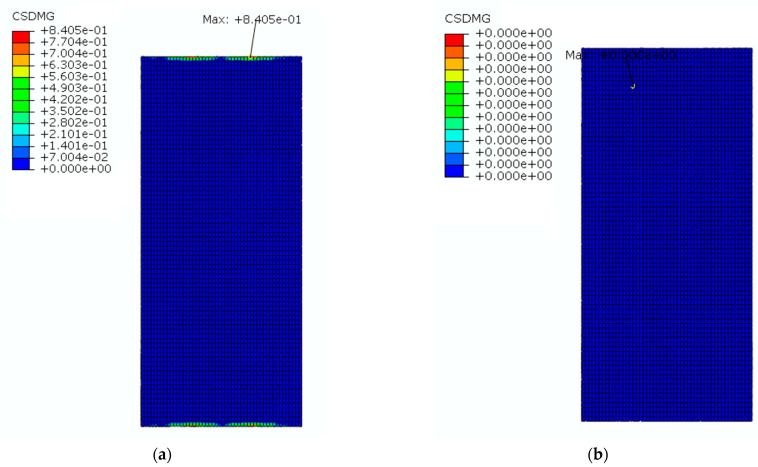
The cloud diagram for the maximum damage of the interlaminar interface: (**a**) before coating; (**b**) after coating.

Figure 29 shows a cloud diagram for the maximum damage of the interlaminar interface before and after coating. In Figure 29, it can be seen that the interlaminar interface was first damaged from the middle of its lateral edge. Before applying the reflective coating, the maximum value of the structure’s CSDMG under natural environmental conditions for one day was 0.84, and the interlaminar interface started to be damaged. After applying the reflective coating, the maximum value of the structure’s CSDMG under natural environmental conditions for one day was 0, and the interlayer interface was not damaged. Therefore, it can be seen that the application of the reflective coating could inhibit the failure of the interlayer interface more effectively.

## 6. Conclusions

In this paper, based on meteorological data and the characteristics of the multi-layer structure of the ballastless track, a refined FEM for the temperature field and temperature effect analysis of the CRTS III slab ballastless track structure on bridges was established. The temperature deformation characteristics and temperature stress distribution of the CRTS III slab ballastless track under natural environmental conditions are investigated. Similarly, the influence of a reflective coating on the structural temperature field and temperature effect were studied. The main findings were as follows:

The reflective coating could significantly reduce the vertical temperature and temperature gradient of the track slab. Under high-temperature conditions in summer, the maximum temperature of the track slab decreased from 47.0 °C to 39.6 °C after the application of a reflective coating, and the maximum vertical temperature gradient of the track slab decreased from 61.5 °C/m to 39.1 °C/m after the application of a reflective coating. However, the effect of the reflective coating on the lateral temperature and temperature gradient of the structure was not significant. 

Under the maximum positive temperature gradient, the slab and the SCC showed a convex spatial deformation pattern: arching in the middle of the structure and sinking in the corner of structure. Under the positive temperature gradient, the peak displacement of the upper arch in the middle of the track slab decreased from 0.814 mm to 0.441 mm with the reflective coating; meanwhile, the peak displacement of the sinking in the track slab corner decreased from 1.240 mm to 0.511 mm after applying the reflective coating. The peak displacement of the upper arch in the middle of the SCC decreased from 0.727 mm to 0.370 mm with the reflective coating; meanwhile, the peak displacement of the sinking in the SCC corner decreased from 1.269 mm to 0.540 mm with the reflective coating. Therefore, the reflective coating significantly reduced the peak displacement of the structural middle and corner of the track slab and SCC under positive temperature gradients.

Under the maximum negative temperature gradient, the slab and the SCC showed a concave deformation pattern: the corner of structure warped up, and the middle area of structure sunk. Under the negative temperature gradient, the peak displacement of sinking in the middle of the track slab decreased from 0.154 mm to 0.090 mm with the reflective coating; meanwhile, the peak displacement of the upward arch of the track slab corner decreased from 0.577 mm to 0.463 mm with the reflective coating. The peak displacement of the sinking in the middle of SCC decreased from 0.180 mm to 0.117 mm with the reflective coating; meanwhile, the peak displacement of the upward arch of the SCC corner decreased from 0.519 mm to 0.406 mm with the reflective coating. Therefore, the reflective coating significantly reduced the peak displacement of the structural middle and corner of the track slab and SCC under negative temperature gradients.

Under the maximum positive temperature gradient, the maximum tensile stress s11 in the transverse direction of the track slab decreased from 2.7 MPa to 1.5 MPa after the reflective coating was applied; the maximum tensile stress s33 in the longitudinal direction of the track slab decreased from 2.2 MPa to 2.0 MPa after the reflective coating was applied. Under the maximum positive temperature gradient, the maximum tensile stress s11 in the transverse direction of the SCC decreased from 3.5 MPa to 1.6 MPa after the reflective coating was applied; the maximum tensile stress s33 in the longitudinal direction of the SCC decreased from 3.2 MPa to 1.5 MPa after the reflective coating was applied. Therefore, the reflective coating could significantly reduce the maximum tensile stress in the transverse direction of the track slab and the maximum tensile stress in the transverse and longitudinal directions of the SCC.

Under natural environmental conditions for one day, the interlayer interface started to be damaged in the middle of the track slab’s lateral edge when the reflective coating was not applied, and the maximum damage value reached 0.84; after the reflective coating was applied, the maximum value of the interlayer interface’s damage initiation variable was less than 1, and the starting damage value of the interlayer interface was not reached yet, so the interlayer interface did not start to be damaged at this time. Therefore, it can be seen that the application of a reflective coating could inhibit the failure of the interlayer interface more effectively.

## Figures and Tables

**Table 1:** Heat transfer material parameters and geometric parameters of the CRTS III slab ballastless track structure.

Structural Layer	Length×Width×Height (mm)	Density (kg/m^3^)	Specific Heat (J/kg·°C)	Thermal Conductivity W/(m·°C)
Track slab	5600 × 2500 × 200	2500	921	2.0
SCC	5600 × 2500 × 90	2500	921	2.0
Base plate	5600 × 2900 × 200	2500	921	2.0

**Table 2:** Thermal analysis model parameters of the CRTS III slab ballastless track structure.

Component	Density (kg/m^3^)	Modulus of Elasticity (MPa)	Poisson Ratio	Expansion Coefficient (10^−5^ °C/m^3^)
Rail	7830	210,000	0.3	1.18
Track slab	2500	36,000	0.3	1.0
SCC	2500	32,500	0.3	1.0
Base plate	2500	31,500	0.3	1.0
Prestressed steel	7800	170,000	0.3	1.18
CRR	7800	170,000	0.3	1.18

**Table 3:** WJ-8-type fastener longitudinal resistance (kN/(m·rail)).

Fastener Type	Under Locomotive	Vehicle under or without Load
WJ-8	$r=\left\{\begin{array}{l} 18.6x\;\;x\leq 2.0\;\mathrm{mm}\\ 37.2\;\;x>2.0\;\mathrm{mm} \end{array}\right.$	$r=\left\{\begin{array}{l} 12.0x\;\;x\leq 2.0\;\mathrm{mm}\\ 24.0\;\;x>2.0\;\mathrm{mm} \end{array}\right.$

## Data Availability

Not applicable.
